# Shining a spotlight on youth involvement in mental health research: Challenges and innovations

**DOI:** 10.1002/jcv2.12285

**Published:** 2024-11-22

**Authors:** Maria Michail

**Affiliations:** ^1^ Institute for Mental Health School of Psychology University of Birmingham Birmingham UK; ^2^ Centre for Youth Mental Health The University of Melbourne Melbourne VIC Australia

**Keywords:** mental health, participatory research, patient and public involvement, young people

## Abstract

Despite progress in youth involvement in mental health research, considerable gaps remain in our understanding, conceptualisation, and implementation of involving children and young people in this field. This Editorial Perspective reflects on how these gaps present challenges to our research practices and often serve as barriers to meaningfully involving youth voices and experiences into the research process. We take a critical perspective to recent advances in the field of youth involvement in mental health research, reflected by the studies included in this special issue, and highlight examples of good practice paving the way for more equitable and inclusive approaches. Meaningful progress in mental health research relies on the active involvement of young people. Researchers, practitioners and policymakers have an ethical and moral responsibility to create a safe and inclusive environment that empowers young people to participate in research that impacts their lives, care, and overall quality of life.

**Key points**

- There remain significant gaps in how we conceptualise and practice youth involvement in mental health research, including underrepresentation of minoritised communities and lack of systematic reporting and evaluation of involvement practices.
- Recent research highlights innovative and inclusive approaches to youth involvement across a range of settings (e.g., educational settings) and populations (e.g., children, neurodivergent adolescent girls).
- Recent research findings, however, also raise questions about transparency, replicability and translatability of youth mental health research informed by patient and public involvement (PPI).
- Challenges in the documentation, reporting and evaluation of youth involvement practices in mental health research could be addressed through the adoption of established guidelines and frameworks aimed at enhancing the quality and transparency of PPI reporting.
- Researchers, practitioners and policymakers have an ethical and moral responsibility to create a safe and inclusive environment that empowers young people to participate in research that impacts their lives, care, and overall quality of life.

The involvement of children and young people in the design, delivery and implementation of mental health research has received widespread attention over the last decade. Child participation is acknowledged as a fundamental right by the United Nations Convention on the Rights of the Child (UNCRC). Many funding bodies now mandate the involvement of patients and public in the planning and delivery of research. There is a proliferation of recommendations, standards (e.g. UK Standards for public involvement in research) and toolkits providing guidance and practical tips to researchers to ensure the meaningful involvement of public, including young people, in mental health research (e.g., McPin Young People's Advisory Group, 2021).

Despite progress, however, there remain significant gaps in how we understand, conceptualise and practice the involvement of children and young people in mental health research. These gaps pose significant challenges to our research praxis and in many cases act as barriers to fully integrating youth voices and experiences into the research process. There are specific groups of young people, for example, those with lived and living experience of self‐harm or suicidal experiences, who are systematically excluded from involvement practices due to perceived safety concerns (Michail et al., 2023). Similarly, young people from underserved and minoritised communities (Denford et al., 2024) are significantly underrepresented in involvement opportunities due to being “*hard‐to‐reach*”, or, as Lightbody (2017) put it, “*easy‐to‐ignore*”. Lack of representation of diverse youth voices could limit the generalisability of research findings, lead to policies that do not fully address the unique needs of underserved communities, thereby, perpetuating existing health inequalities (Denford et al., 2024). An additional challenge arises from the lack of systematic reporting and evaluation of involvement practices making it difficult to advance learning and improve practice. This is particularly pertinent when considering the potentially adverse effects of involving those with lived experience in mental health research (Morgan, 2024).

The current special issue of JCPP Advances focuses on the role of participatory research in involving youth voices in the co‐creation of knowledge and practice across all stages of mental health research. The studies included in this special issue reflect a concerted effort by the scientific community to address some of the pressing challenges in youth involvement paving the way for more equitable and inclusive practice.

Bartnick et al. (2024) highlight different approaches in involving younger children (aged 6–12) in mental health research including child‐friendly communication, age‐appropriate interactive methods to create a safe, supportive and effective environment for involvement. The authors share learnings and offer recommendations to researchers for enhancing involvement practices among children, whose voices are often neglected in research.

Khawaja et al. (2024) reflect on the benefits and challenges of youth participatory action research (YPAR) in schools by drawing upon a case study, breaking the silence (BtS). BtS forms part of a series of YPAR projects, co‐facilitated by educational psychologists, aiming at improving existing educational structures by placing the voice of students at the centre. The authors reflect on the challenges associated with facilitating YPAR and offer insights into how to mitigate such challenges ensuring YPAR has the potential to be an empowering approach. The study has important implications for the use of YPAR in educational settings by a range of professionals (e.g., teachers, educational psychologists). Importantly, this work contributes to the scientific dialogue about the role of YPAR in enhancing student agency, power sharing and collaborative learning.

Jones et al. (2024) conducted the first realist review of international literature on co‐production in mental health services for youth. This approach is unique in that it considers the involvement of stakeholders (in this case, young people) throughout the review process, ensuring that the findings are relevant and applicable to real‐world contexts. The adoption of realist review by Jones et al. (2024) led to the development of a programme theory about *how*, *why* and *for whom* co‐production methods in youth mental health services work. The theory highlights the role of supportive organisational culture (particularly for those from minoritised communities), resourcing and transparency about power sharing and the limits for change. This work provides a deeper and more nuanced understanding of the generative mechanisms (processes, structures) of co‐production in mental health services for youth with practical implications for policymakers and practitioners.

McKinney et al. (2024) investigate the role of camouflaging in neurodivergent and neurotypical adolescent girls in a study co‐produced with adult neurodivergent women. The authors describe how they worked collaboratively with 15 neurodivergent women to highlight the use of camouflaging by girls as a key research priority focussing on its links to poor mental health. The project was then co‐designed to address this research priority. Despite increasing awareness of the importance of including neurodivergent individuals in research, many projects still do not fully integrate neurodivergent voices throughout the research process (Pells, 2022). The work by McKinney et al. (2024) offers important insights into the feasibility of meaningfully engaging with neurodivergent populations and the value of inclusive involvement practice in this space.

Bakermans‐Kranenburg and van IJzendoorn (2024) raise important questions about transparency, replicability and translatability of youth mental health research informed by patient and public involvement (PPI). The authors reviewed PPI studies included in two systematic reviews and coded data in relation to PPI characteristics (e.g., yes/no: PPI as co‐researchers), transparency (e.g., yes/no: information on PPI recruitment; sample size; age; gender; SES/Ethnicity), and translation (e.g., yes/no: translation to policy and/or practice). The authors highlight examples of good practice whereby studies systematically reported the range and type of PPI roles across the research cycle. There was, however, less clarity about PPI recruitment and sampling strategies which raises questions about representativeness in PPI practices and challenges in ensuring diversity and inclusivity. The authors also highlight that the translatability of research (informed by PPI) was limited to the development and dissemination of recommendations for policy and/or practice (sometimes in the absence of replicable findings). The study by Bakermans‐Kranenburg and van IJzendoorn (2024) highlights key challenges in the documentation, reporting and evaluation of involvement practices in youth mental health research. Such challenges could be addressed through the use of guidelines developed to facilitate the effective and transparent reporting of PPI in research. For example, the GRIPP2 reporting checklists (Staniszewska et al., 2017) offers a structured framework for reporting PPI activities including aims, methods, context, stages of involvement, outcomes thereby enhancing the quality and transparency of PPI reporting. The Public Involvement Impact Assessment Framework (PiiAF; Popay et al., 2014) is designed to help researchers reflect on their PPI approach, values and practical issues as a way of assessing the impacts of involving members of the public in their research. It is important to highlight that PPI is a dynamic social process comprising of many interconnected elements related to context (e.g. stakeholders, type of research), processes (e.g., how stakeholders are involved), structures (e.g., governance structures), values (e.g., inclusivity, cultural sensitivity, power sharing). Due to the evolving nature of PPI, its impact may not be immediately apparent, or it might change over time. This is particularly relevant in research areas such as youth mental health where researchers need to be attuned to the complexities and sensitivities of working with potentially vulnerable children and young people (and the impact of this on the involvement process).

This special issue of JCPP Advances shines a spotlight on the critical role that youth voices play in informing and shaping the future of mental health research. By showcasing examples of co‐designed studies and participatory methods, the issue advances our understanding of innovative approaches to involving children and young people as active partners in research and encourages reflective practice as we strive for more inclusive, equitable and impactful research outcomes.

## AUTHOR CONTRIBUTION

**Maria Michail:** Conceptualization; Writing ‐ original draft; Writing ‐ review and editing.

## CONFLICT OF INTEREST STATEMENT

The author has declared that they have no competing or potential conflicts of interest.

## ETHICAL CONSIDERATIONS

None.

## Data Availability

Data sharing not applicable to this article as no datasets were generated or analysed.
